# A gene mutation-based risk model for prognostic prediction in liver metastases

**DOI:** 10.1186/s12864-023-09595-9

**Published:** 2023-08-26

**Authors:** Bingran Yu, Ning Zhang, Yun Feng, Weiqi Xu, Ti Zhang, Lu Wang

**Affiliations:** Department of Hepatic Surgery, Shanghai Cancer Center, Shanghai Medical College, Fudan University, No. 270 Dongan Road, Shanghai, 200032 People’s Republic of China

**Keywords:** Gene mutation, Risk model, Liver metastasis, Tumor microenvironment

## Abstract

**Background:**

Liver metastasis is the major challenge in the treatment for malignant tumors. Genomic profiling is increasingly used in the diagnosis, treatment and prediction of prognosis in malignancies. In this study, we constructed a gene mutation-based risk model to predict the survival of liver metastases.

**Method:**

We identified the gene mutations associated with survival and constructed the risk model in the training cohort including 800 patients with liver metastases from Memorial Sloan-Kettering Cancer Center (MSKCC) dataset. Other 794 patients with liver metastases were collected from 4 cohorts for validation. Furthermore, the analyses of tumor microenvironment (TME) and somatic mutations were performed on 51 patients with breast cancer liver metastases (BCLM) who had both somatic mutation data and RNA-sequencing data.

**Results:**

A gene mutation-based risk model involved 10 genes was constructed to divide patients with liver metastases into the high- and low-risk groups. Patients in the low-risk group had a longer survival time compared to those in the high-risk group, which was observed in both training and validation cohorts. The analyses of TME in BCLM showed that the low-risk group exhibited more immune infiltration than the high-risk group. Furthermore, the mutation signatures of the high-risk group were completely different from those of the low-risk group in patients with BCLM.

**Conclusions:**

The gene mutation-based risk model constructed in our study exhibited the reliable ability of predicting the prognosis in liver metastases. The difference of TME and somatic mutations among BCLM patients with different risk score can guide the further research and treatment decisions for liver metastases.

**Supplementary Information:**

The online version contains supplementary material available at 10.1186/s12864-023-09595-9.

## Introduction

Liver metastases are tumors which have spread from primary sites of cancers to liver and are the major cause of treatment failure and mortality in malignant tumors. The most common cancer which metastasizes to the liver is colorectal cancer, followed by pancreatic cancer, breast cancer, melanoma and lung cancer [1]. Despite variety of treatments including surgery, chemotherapy, targeted therapy and immune checkpoint inhibitors were used in the treatment for liver metastases, poor response and rapid recurrence of tumors were often observed in liver [2]. It is necessary to construct a model which can predict the prognosis of liver metastases and guide the clinical treatment.

Genomic profiling is increasingly used to identify the pathogenic genes, select the targeted treatments and develop prognostic biomarkers [3]. In a phase II non-randomized clinical trial of Hayashi et al*.* [4], molecularly targeted therapies based on profiling gene expressions and gene alterations by next-generation sequencing (NGS) were applied in the treatment for cancer of unknown primary site and were associated with a favorable survival outcome. The study of Goss et al*.* [5] used NGS to identify patients with lung squamous cell carcinoma who would derive additional benefit from treatment with afatinib and found ERBB and HER2 mutations could act as predictive markers for afatinib treatment. Furthermore, Long et al*.* [6] constructed and validated a gene mutation-based gene set to predict the prognosis of patients treated with immune checkpoint therapy.

In the present study, we integrated the cohorts of liver metastases to construct and validate a novel risk model based on gene mutations to predict the prognosis of patients with liver metastases. Additionally, we further analyzed the expression profiles of patients with different risk scores.

## Materials and methods

### Study population

The mutation data and clinical information of the training, primary liver cancer (PLC) and validation cohorts were obtained from the cBioPortal database (https://www.cbioportal.org). In the training cohort, both mutation data and clinical information were available for 800 patients with liver metastases from Memorial Sloan Kettering Cancer Center (MSKCC) database reported by Zehir et al*.* [7]. In the validation cohort, both mutation data and clinical information were available for 136 patients with liver metastases from the cohort of Samstein et al*.* [8], 312 patients with liver metastases from the cohort of Yaeger et al*.* [9], and 198 patients with liver metastases from the cohort of Pleasance et al*.* [10]. The PLC cohort, including 138 patients with PLC from MSKCC database reported by Zehir et al*.* [7], 107 patients with intrahepatic cholangiocarcinoma (ICC) from the cohort of Lowery et al*.* [11], 114 patients with hepatocellular carcinoma (HCC) from the cohort of Ng et al*.* [12], 61 patients with combined HCC and ICC from the cohort of Xue et al*.* [13], and 357 patients with HCC or ICC from The Cancer Genome Altas (TCGA) database, was also used for analysis. The clinical information of each sample enrolled in the analysis were shown in Additional file 1: Fig. S1 and Additional file 2: Table S1-S3.

One hundred forty-eight patients with liver metastasis treated in Department of Hepatic Surgery of Fudan University Shanghai Cancer Center (FUSCC) were enrolled in the validation cohort. All patients provided their informed consents. The study was approved by the Institutional Review Board of FUSCC. The clinical information was collected retrospectively and shown in Additional file 2: Table S2. A customed-designed genetic panel, comprising a hybridization-capture-based assay of 484 genes, was used to identify the mutant genes. The genes used in sequencing were listed in Additional file 2: Table S4. Patient’s genomic DNA was extracted from tumor tissues. The libraries were pooled and sequenced using an Illumina HiSeq X TEN platform (Illumina Inc., San Diego, CA, USA). Data were collected using Illumina Real Time Analysis (RTA) and assembled to FASTQ files using Illumina Bcl2Fastq2. Then, the high-quality reads were mapped to the hg19 version of the human reference genome (GRCh37) using BWA aligner with the BWA-MEM algorithm and default parameters. The Genome Analysis ToolKit was used to locally realign the BAM files at intervals with mismatched indels and recalibrate the base quality scores of the reads in the BAM files. Somatic mutations were called from the tissue BAM files using GATK4 Mutect2 with the default parameters. Finally, the variants and annotation results were transferred into Excel spreadsheets for further analyses.

In the cohort of Pleasance et al*.* [10], 51 patients with breast cancer liver metastases (BCLM) were included into the transcriptomic cohort, whose RNA-sequencing data were acquired from UCSC Xena (https://xenabrowser.net/datapages/). The clinical data of each sample in the transcriptomic cohort were shown in Additional file 1: Fig. S1 and Additional file 2: Table S5.

### Study design

**Fig. 1:**
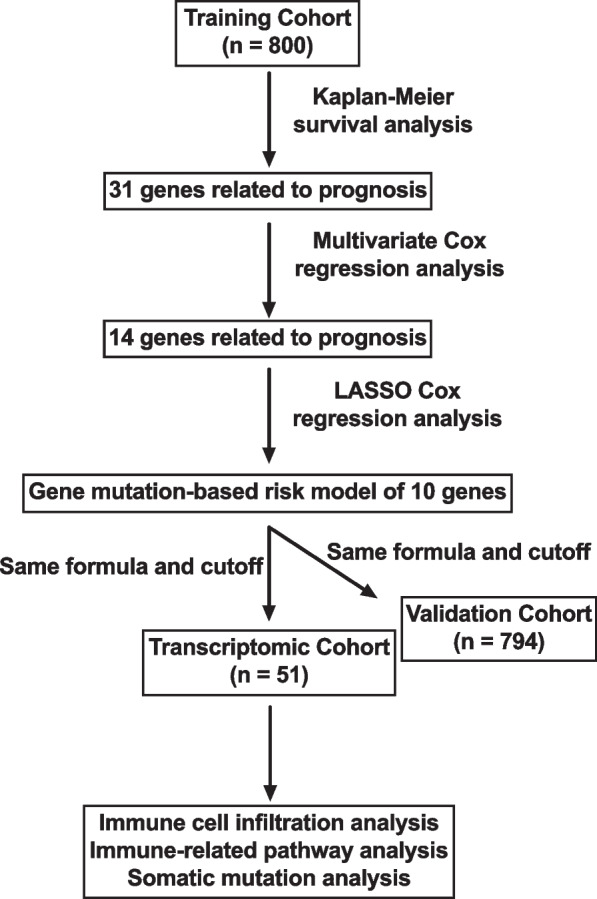
The workflow of the present study

Figure 1 and Additional file 1: Fig. S1 summarized the analysis process and cohorts used in the study that included training, validation, PLC and transcriptomic cohorts. First, propensity score matching (PSM) method by “MatchIt” R package was used in the training cohort to balance potentially confounding factors, including sex and cancer types, between the mutant-type and wild-type status of each gene in the MSKCC-IMPACT panel (Additional file 2: Table S6). Then survival data were compared between the mutant-type and wild-type status of each gene using Kaplan–Meier method in univariate analysis. Genes with *P* < 0.05 in univariate analysis were candidates for entry into multivariate Cox regression analysis. Finally, LASSO Cox regression analysis using “glmnet” R package was applied to identify prognostic genes from genes with *P* < 0.05 in multivariate analysis and constructed a gene mutation-based risk model. The risk score was calculated according to the formula: Risk score = ∑ C_i_ X_i_ where C_i_ is the coefficient of each prognostic gene and X_i_ is the expression value of each prognostic gene. X_i_ is 0 when the prognostic gene is wild type, and 1 when the prognostic gene is mutant type. X-tile 3.6.1 software was applied to determine the best cutoff for classifying patients into high- and low-risk groups [14]. The same formula and cutoff were used for the validation and transcriptomic cohorts.

### Tumor microenvironment analyses

Tumor microenvironment (TME) analyses were compared between the high- and low-risk groups based on the RNA-sequencing data of 51 patients with BCLM in the transcriptomic cohort. The genes used to quantify the enrichment levels of immune cell types and immune-related pathways were acquired from Long et al*.* [6]. The “GSVA” R package based on single-sample gene set enrichment analysis (ssGSEA) method was used to quantify the enrichment levels of these immune cells and immune-related pathways in each sample [15]. The enrichment scores in ssGSEA analysis were normalized to unity distribution, for which the minimal score is zero and maximal score is one. All immune-related signatures used in the TME analyses were shown in Additional file 2: Table S7 [6].

### Drug sensitivity analysis

The “oncoPredict” package was used to assess the drug sensitivity of 198 chemotherapeutic drugs between patients in the high- and low-risk groups.

### Functional enrichment analysis

Differential expression genes (DEGs) between the high- and low-risk groups were identified using the “limma” R package with a fold change of 2 and an adjusted *P*-value < 0.05. Functional enrichment analysis of DEGs were performed based on Gene Oncology (GO) and Kyoto Encyclopedia of Genes and Genomes (KEGG) using “clusterProfiler” R package [16].

### Somatic mutation analyses

The “maftools” R package was used to analyze the difference of somatic mutations between the high- and low-risk groups in the transcriptomic cohort.

### Statistical analyses

Statistical analyses were performed using R 4.2.2 and GraphPad Prism (Version 9.4.1). Cox regression models were applied to assess the independent prognostic value of the risk model. To evaluate the accuracy of the risk model, the receiver operating characteristic (ROC) curves were generated by the “timeROC” package, and the Area Under Curve (AUC) and C-index were compared with other risk factors. The calibration curve was constructed using “rms” R package to explore the predictive accuracy of the risk model. The Wilcoxon test was conducted to compare differences between two groups. *P*-values were two-sided with *P* < 0.05 considered significant.

## Results

### Construction of the gene mutation-based risk model

We performed PSM analysis and compared the survival outcomes between the mutant-type and wild-type status of each gene in the training cohort. The mutations of 31 genes were found to be associated with survival and the multivariate analysis identified 14 prognostic gene mutations in patients with liver metastasis (Additional file 2: Table S8). The LASSO regression analysis was applied to identified 10 prognostic gene mutations and their coefficients. The risk score was constructed as follow: Risk score = *APC* × (-0.447543265) + *B2M* × (1.765754185) + *FANCA* × (0.231443411) + *FAT1* × (-0.238096394) + *IRS1* × (1.223490281) + *MAP3K13* × (0.000988332) + *NTRK1* × (0.840546061) + *STK11* × (0.470490681) + *TP53* × (0.198123143) + *YES1* × (0.102246259). In the formula, the mutant gene status was coded as 1, and the wild gene status was coded as 0. The optimal cutoff value was zero. The patients with risk score ≥ 0 were divided to the high-risk group and those with risk score < 0 were divided to the low-risk group.

### Comparison of survival between the high- and low-risk groups

**Fig. 2:**
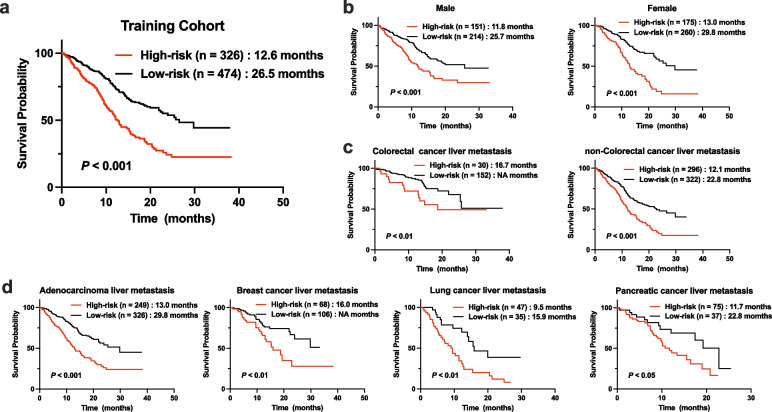
Survival analysis of gene mutation-based risk model in the training cohort and different subgroups. **a** Survival analysis of gene mutation-based risk model in the training cohort. **b** Survival analysis of gene mutation-based risk model in male and female patients. **c** Survival analysis of gene mutation-based risk model in Colorectal cancer liver metastasis and non-Colorectal cancer liver metastasis. **d** Survival analysis of gene mutation-based risk model in different cancer types (adenocarcinoma liver metastasis, breast cancer liver metastasis, lung cancer liver metastasis and pancreatic cancer liver metastasis)

In the training cohort, patients in the low-risk group had a longer overall survival (OS) than those in the high-risk group (26.5 months vs. 12.6 months, *P* < 0.001; Fig. 2a). Subgroup analysis based on sex and cancer types also indicated that the longer OS were observed in the patients with low risk scores compared to those with high risk scores (Fig. 2b-d).

**Fig. 3:**
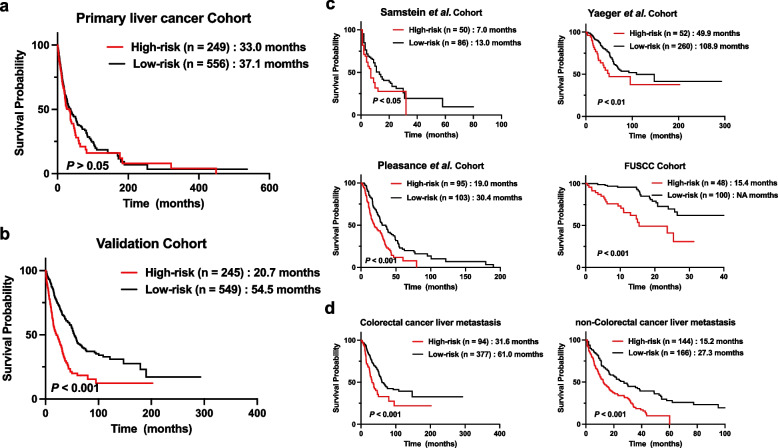
Survival analysis of gene mutation-based risk model in the primary liver cancer, validation cohorts and different subgroups. **a** Survival analysis of gene mutation-based risk model in the primary liver cancer cohort. **b** Survival analysis of gene mutation-based risk model in the validation cohort. **c** Survival analysis of gene mutation-based risk model in different cohorts of validation cohort. **d** Survival analysis of gene mutation-based risk model in Colorectal cancer liver metastasis and non-Colorectal cancer liver metastasis of validation cohort

We also compared the OS of PLC cohort to investigate whether the risk model was also applicable to patients with PLC. There was no significant difference in survival between patients in the high- and low-risk groups (33.0 months vs. 37.1 months, *P* > 0.05; Fig. 3a).

The same formula and cutoff were also used in the validation cohort to evaluate the risk model. In the validation cohort, patients in the low-risk group exhibited a better OS than those in the high-risk group (54.5 months vs. 20.7 months, *P* < 0.001; Fig. 3b). Furthermore, subgroup analysis based on different cohorts and cancer types showed that patients in the low-risk group had a longer survival time than those in the high-risk group (Fig. 3c-d).

### Analyses of the predictive value of the gene mutation-based risk model

The AUC of the gene mutation-based risk model in the training cohort was 0.67 at 1 year, 0.64 at 2 years and 0.61 at 3 years (Additional file 1: Fig. S2a); the AUC in the validation cohort was 0.86 at 1 year, 0.73 at 2 years and 0.67 at 3 years (Additional file 1: Fig. S2b). The AUC for different cancer types of training cohorts (Additional file 1: Fig. S2c) and different cohorts of validation cohorts (Additional file 1: Fig. S2d) were also assessed. The calibration curve of the risk model was constructed and showed good agreement between the observations and the predictions in the training (Additional file 1: Fig. S3a) and validation cohorts (Additional file 1: Fig. S3b).

To further analyze the predictive value of the gene mutation-based risk model, the multivariate Cox analysis of sex, age, tumor mutation burden (TMB), primary cancer types and risk model were performed. The risk score and primary cancer types were the independent prognostic factors for patients with liver metastasis in the training cohort (Additional file 1: Fig. S3c), which was also observed in the validation cohort (Additional file 1: Fig. S3d).

We used C-index to compared the performance of the gene mutation-based risk model with 10 genes included in the risk model and found that the risk model exhibited more excellent predictive power than 10 separate genes either in the training cohort or in the validation cohort.

### TME analyses between the high- and low-risk groups

**Fig. 4:**
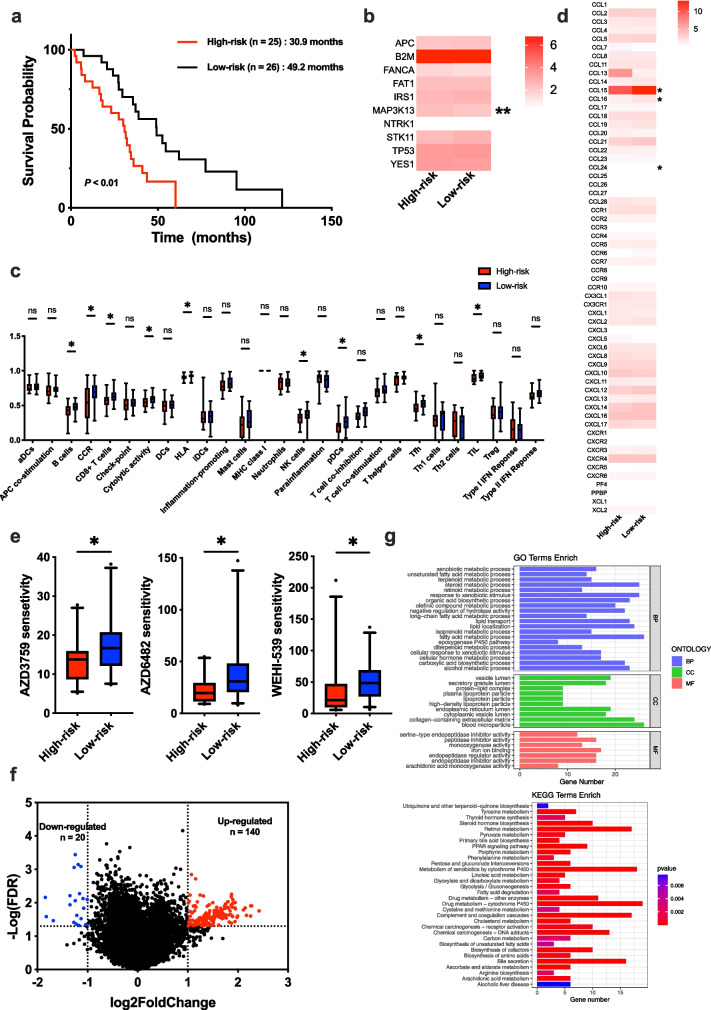
Tumor microenvironment analysis between the high- and low-risk groups in patients with breast cancer liver metastases (BCLM). **a** Survival analysis between the high- and low-risk groups in patients with BCLM. **b** mRNA expression profiles analysis of 10 genes included in the risk model between the high- and low-risk groups in patients with BCLM. **c** Comparison of the immune infiltration estimated by the ssGSEA method based on RNA-sequencing data between the high and low-risk groups in patients with BCLM. **d** Comparison of genes associated with chemokines between the high and low-risk groups in patients with BCLM. **e** Drug sensitivity analysis between the high- and low-risk groups in patients with BCLM. **f** Volcano plot of differential expression genes (DEGs) between the high and low-risk groups in patients with BCLM. The high-risk group was the controlled group. **G** GO and KEGG functional enrichment analysis of DEGs between the high and low-risk groups in patients with BCLM

We explored the difference of TME based on the RNA-sequencing data of transcriptomic cohort, in which 51 patients with BCLM were classified into the high- and low-risk groups using the same formula and cutoff (Additional file 1: Fig. S1d). A better OS was observed in the low-risk group compared to the high-risk group in the transcriptomic cohort (49.2 months vs. 30.9 months, *P* < 0.01; Fig. 4a). Among 10 genes included in the risk model, *MAP3K13* exhibited a higher expression level in the high-risk group than in the low-risk group (Fig. 4b). We evaluated the enrichment scores of immune cells infiltration and immune-related pathways between the high- and low-risk groups using ssGSEA method. More immune cell infiltrations, including B cells, CD8^+^ T cells, Natural Killer (NK) cells, plasmacytoid dendritic cells (pDCs), follicular helper T cells (Tfh) and tumor infiltrating lymphocytes (TILs) were found in the low-risk group (Fig. 4c). Furthermore, the low-risk group also show the higher activity of pathways related to C-C chemokine receptor (CCR), cytolytic activity and human leukocyte antigen (HLA) than the high-risk group (Fig. 4c). The analysis of the expression levels of genes associated with chemokines indicated that the high-risk group expressed the higher levels of CCL15, CCL16 and CCL24 than the low-risk group (Fig. 4d). We assessed the drug sensitivity of the high- and low-risk populations to chemotherapeutic drugs and found that the low-risk group had the higher sensitivity to AZD3759, AZD 6482 and WEHI-539 (Fig. 4e).

We performed the analysis of DEGs between the high- and low-risk groups in the transcriptomic cohort. Compared to the high-risk group, a total of 160 DEGs were identified with 140 up-regulated genes and 20 down-regulated genes in the low-risk group (Fig. 4f). The function enrichment analysis of DEGs based on GO and KEGG databases showed that DEGs between the high- and low-risk groups involved in pathways associated with lipid metabolism such as fatty acid metabolism, lipid transport, lipid localization and lipoprotein particle in GO, and PPAR signaling pathway, fatty acid degradation and cholesterol metabolism in KEGG (Fig. 4g, Additional file 2: Table S9).

### Somatic mutation analyses between the high- and low-risk groups

**Fig. 5:**
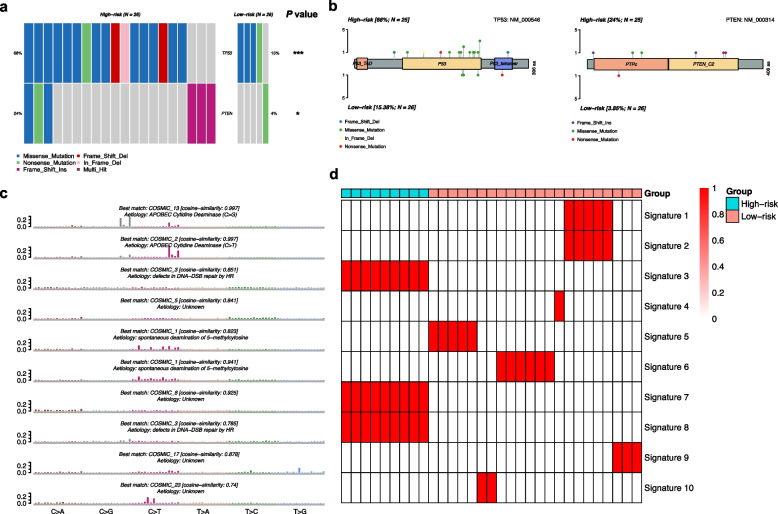
Somatic mutation analysis between the high and low-risk groups in patients with breast cancer liver metastases (BCLM). **a** Comparison of gene mutations between the high and low-risk groups in patients with BCLM. **b** Lillipop plot of *TP53* and *PTEN* mutation between the high and low-risk groups in patients with BCLM. **c** Mutation signatures of patients with BCLM. **d** Comparison of mutation signatures between the high and low-risk groups in patients with BCLM

Somatic mutation analyses were performed in the transcriptomic cohort*.* Besides *TP53*, the high-risk group also exhibited higher frequencies of gene mutations in *PTEN* than the low-risk group (Fig. 5a-b). We analyzed the mutation signatures and compared the extracted mutation signatures against the Catalogue of Somatic Mutations in Cancer (COSMIC) by cosine similarity. 10 mutation signatures were identified. Three mutation signatures that associated with defects in DNA-DSB repair by HR were observed in the high-risk group, while other seven mutation signatures were found in the low-risk group (Fig. 5c-d).

## Discussion

Genomic profiling has been increasingly used in constructing the risk model to predict either the response to immunotherapy or the prognosis of patients with malignant tumors [6, 17–19]. To the best of our knowledge, the present multicohort study was the first one that constructed and validated a prognostic risk model based on gene mutations to predict the survival outcomes of patients with liver metastases. In our study, 10 genes mutations were identified as the prognostic genes mutations. Among them, most of gene mutations have been reported to be associated with the occurrence and progression of cancers. The mutations of *TP53* can impair antitumor activity and confer mutant *p53* protein oncogenic properties [20]. *FAT1* exhibited complexity in modulating tumorigenesis and acted as tumor promoter or suppressor depending on tumor types [21, 22]. Furthermore, tumors without mutations of *APC* were reported to be carry a worse prognosis than single *APC* mutation tumors in patients with colorectal cancers [23]. In our risk model, the coefficient of *FAT1* and *APC* was less than zero, demonstrated the mutations of these two genes suppressed the progression of tumors in patients with liver metastases.

Patients were divided into the high- and low-risk group according to the risk scores and a better OS was observed in the low-risk group compared to the high-risk group. However, it should be mentioned that of patients with colorectal cancer liver metastasis (CRCLM) or other cancer liver metastasis (OCLM), only approximately 20% were divided into the high-risk group. OCLM contained various uncommon cancers which had difference tumor characteristics, thus leading to less patients in the high-risk group. For patients with CRCLM, the reason of the imbalanced distribution could be due to the high frequency of *APC* mutations in CRC and the negative coefficient of *APC* in the risk score [24]. Despite of the different distribution among the different liver metastases, better survival was observed in all low-risk groups. In patients with PLC, there was no significant difference of survival outcomes between the high- and low-risk groups, indicated the risk model wasn’t applicable to PLC. Furthermore, the multivariate analysis indicated that the risk score was also the prognostic factor of patients with liver metastases. We also compared predictive accuracy of the risk model with that of 10 individual gene mutations and a higher C-index of risk model was observed. In summary, based on these analyses and results, our gene mutation-based risk model was reliable in the prediction of survival of patients with liver metastases.

We explored the impact of 10 gene mutations included in the risk model on their gene expression levels. In the patients with BCLM, patients in the high-risk group had a significantly higher expression level of *MAP3K13*. High *MAP3K13* expression was reported to be correlated with poor prognosis in breast cancers, which might be one of the reasons that a shorter survival time was observed in the high-risk group of our study [25].

We used the transcriptomic data of a BCLM cohort to explore the difference of TME between the high- and low-risk groups. We found that the low-risk group had a higher infiltration of immune cells, such as a higher level of B cells, CD8^+^ T cells, NK cells, Tfh and TILs. These immune cells exhibit antitumor effect in the development and progression of tumor [26–28]. It was reported that the depletion of these immune cells was associated with low responses to immunotherapy, thus leading to poor prognosis [29–32]. However, the low-risk group also had more pDCs which impeding T cell-mediated cytotoxicity, and a higher expression level of CCL15, CCL16 and CCL24 that were associated with clinical progression [1, 33]. Therefore, immune cells and chemokines with antitumor or protumor effects were both play a crucial part in the liver metastasis. Furthermore, we found that patients in the low-risk group have a higher response to chemotherapeutic drugs, such as AZD3759 which is a novel epidermal growth factor receptor (EGFR) tyrosine kinase inhibitor (TKI) and produced promising antitumor effect on non-small cell lung cancer [34]. In addition to AZD3759, AZD6482 (*PI3Kβ* inhibitor) and WEHI-539 (a *BCL2L1* inhibitor) were also reported to have potential antitumor effect. Despite these chemotherapeutic drugs have not been widely used, the results might provide guidance for further studies on treatments of patients with BCLM [35, 36].

In current study, the function enrichment analysis showed that DEGs between the high- and low-risk groups were enriched in pathways associated with lipid metabolism. It was reported that lipid metabolism exhibited vital effect by supporting proliferation, survival, migration, invasion and metastases of cancer cells during tumor progression [37]. The high rates of fatty acid oxidation were associated with the high potential metastases of triple-negative breast cancers [38]. Therefore, our study indicated that fatty acid metabolism play a vital role in prognosis of patients with BCLM in the high- and low-risk groups. Furthermore, *PPAR* modulates the lipid homeostasis in liver and the blockage of *PPARγ* can suppress breast cancer progression [39, 40]. The enrichment of *PPAR* pathway was also observed between the high- and low-risk groups in our study, confirmed the important role of *PPAR* in BCLM.

In the analyses of somatic mutation, higher frequencies of mutations in *TP53* and *PTEN* were observed in the high-risk patients with BCLM. *PTEN* is one of the most frequently mutated human tumor suppressor genes and the breast carcinogenesis is potentially associated with *PTEN* loss of activity owing to *PTEN* mutation [41]. Besides, patients in the high-risk group also had the completely different mutation signatures with those in the low-risk group, which might lead to the difference of survival in patients with liver metastases.

This study had several limitations that should be considered. First, this was a study including various liver metastases. Although PSM analysis was performed to balance the bias, some potential factors might still influence the results due to the lack of complete clinical data. Second, despite the risk model exhibited the reliable ability of predicting the survival outcomes in patients with liver metastases, the accuracy was not high enough. Then, biological insight into the basis for the prognostic significance was obtained through analyses of a small (*n* = 51) cohort of BCLM samples. Different biological characteristics can be observed among different cancer types. Therefore, analyses of various types of liver metastases were needed to verify the conclusions which were obtained from analyses of BCLM patients. Finally, the in vivo and in vitro functional experiments were needed to verify the difference of TME between the high- and low-risk groups and to investigate the molecular mechanism underlying the influence of each gene on liver metastases.

## Conclusion

This study constructed and validated a gene mutation-based risk model to predict survival time for patients with liver metastases. The analyses of TME demonstrated that the patients with BCLM in the low-risk group had more immune cell infiltrations than those in the high-risk group. Furthermore, lipid metabolism played a crucial role in BCLM. Our study also revealed distinct mutation signatures for the high- and low-risk groups. Further studies were needed to verify the predictive ability of our gene mutation-based risk model and explore the biological characteristics of patients with different risk scores.

### Supplementary Information

**Additional file 1:  Fig. S1.** The summary of patients included in the training (a), validation (b) and primary liver cancer (c) and transcriptomic (d) cohorts. **Fig. S2.** Assessment of the predictive performance of the gene mutation-based risk model. a. Receiver operating characteristic (ROC) curve of the training cohort. b. ROC curve of the validation cohort. c. Area under the ROC curve (AUC) for different cancer types in the training cohort. d. AUC for different cohorts in the validation cohort. **Fig. S3.** Assessment of the predictive accuracy of the gene mutation-based risk model. a. Calibration curve for assessing the predictive accuracy of the gene mutation-based risk model in the training cohort. The gray line represents ideal performance. The red line represents actual performance. b. Calibration curve for assessing the predictive accuracy of the gene mutation-based risk model in the validation cohort. The gray line represents ideal performance. The red line represents actual performance. c. Univariate and multivariate Cox regression analyses of gene mutation-based risk model in the training cohort. d. Univariate and multivariate Cox regression analyses of gene mutation-based risk model in the validation cohort. e. Comparison of C-indexes between the gene mutation-based risk model and 10 gene mutations included in the risk model in the training cohort. f. Comparison of C-indexes between the gene mutation-based risk model and 10 gene mutations included in the risk model in the validation cohort.

**Additional file 2: Table S1.** The clinical data for each sample used in the training cohort. **Table S2.** The clinical data for each sample used in the validation cohort. **Table S3.** The clinical data for each sample used in the primary liver cancer cohort. **Table S4.** Gene list for FUSCC. **Table S5.** The clinical data for breast cancer liver metastases in the cohort of Pleasance et al. **Table S6.** Gene list for MSKCC. **Table S7.** Immune-related signatures used in the study. **Table S8.** The multivariate Cox regression analysis of gene mutations in patients with liver metastasis. **Table S9.** Functional enrichment analysis of differential expressed genes between the high- and low-risk group in the transcriptomic cohort.

## Data Availability

The data and material were derived from cBioportal, and UCSC Xena, which are publicly available at https://www.cbioportal.org and https://xenabrowser.net/datapages/. The datasets of FUSCC cohort are available from the corresponding author on reasonable request.

## Abbreviations

MSKCC: Memorial Sloan-Kettering Cancer Center dataset
TME: Tumor microenvironment
BCLM: Breast cancer liver metastases
NGS: Next-generation sequencing
PLC: Primary liver cancer
ICC: Intrahepatic cholangiocarcinoma
HCC: Hepatocellular carcinoma
TCGA: The Cancer Genome Altas
FUSCC: Fudan University Shanghai Cancer Center
PSM: Propensity score matching
ssGSEA: Single-sample gene set enrichment analysis
DEG: Differential expression genes
GO: Gene Oncology
KEGG: Kyoto Encyclopedia of Genes and Genomes
ROC: Receiver operating characteristic
AUC: Area Under Curve
OS: Overall survival
TMB: tumor mutation burden
NK: Natural Killer
pDC: Plasmacytoid dendritic cell
Tfh: Follicular helper T cells
TIL: Tumor infiltrating lymphocyte
CCR: C-C chemokine receptor
HLA: Human leukocyte antigen
COSMIC: Catalogue of Somatic Mutations in Cancer
