# Supplementary material for: A gene mutation-based risk model for prognostic prediction in liver metastases
Source: BMC Genomics. 2023 Aug 26;24:489. doi: 10.1186/s12864-023-09595-9 (PMC10463705; doi:10.1186/s12864-023-09595-9)
Supplement: Supplementary file 1 — Additional file 1: Fig. S1. The summary of patients included in the training (a), validation (b) and primary liver cancer (c) and transcriptomic (d) cohorts. Fig. S2. Assessment of the predictive performance of the gene mutation-based risk model. a. Receiver operating characteristic (ROC) curve of the training cohort. b. ROC curve of the validation cohort. c. Area under the ROC curve (AUC) for different cancer types in the training cohort. d. AUC for different cohorts in the validation cohort. Fig. S3. Assessment of the predictive accuracy of the gene mutation-based risk model. a. Calibration curve for assessing the predictive accuracy of the gene mutation-based risk model in the training cohort. The gray line represents ideal performance. The red line represents actual performance. b. Calibration curve for assessing the predictive accuracy of the gene mutation-based risk model in the validation cohort. The gray line represents ideal performance. The red line represents actual performance. c. Univariate and multivariate Cox regression analyses of gene mutation-based risk model in the training cohort. d. Univariate and multivariate Cox regression analyses of gene mutation-based risk model in the validation cohort. e. Comparison of C-indexes between the gene mutation-based risk model and 10 gene mutations included in the risk model in the training cohort. f. Comparison of C-indexes between the gene mutation-based risk model and 10 gene mutations included in the risk model in the validation cohort. [file 12864_2023_9595_MOESM1_ESM.zip › Additional file 1 Fig. S1.pdf]

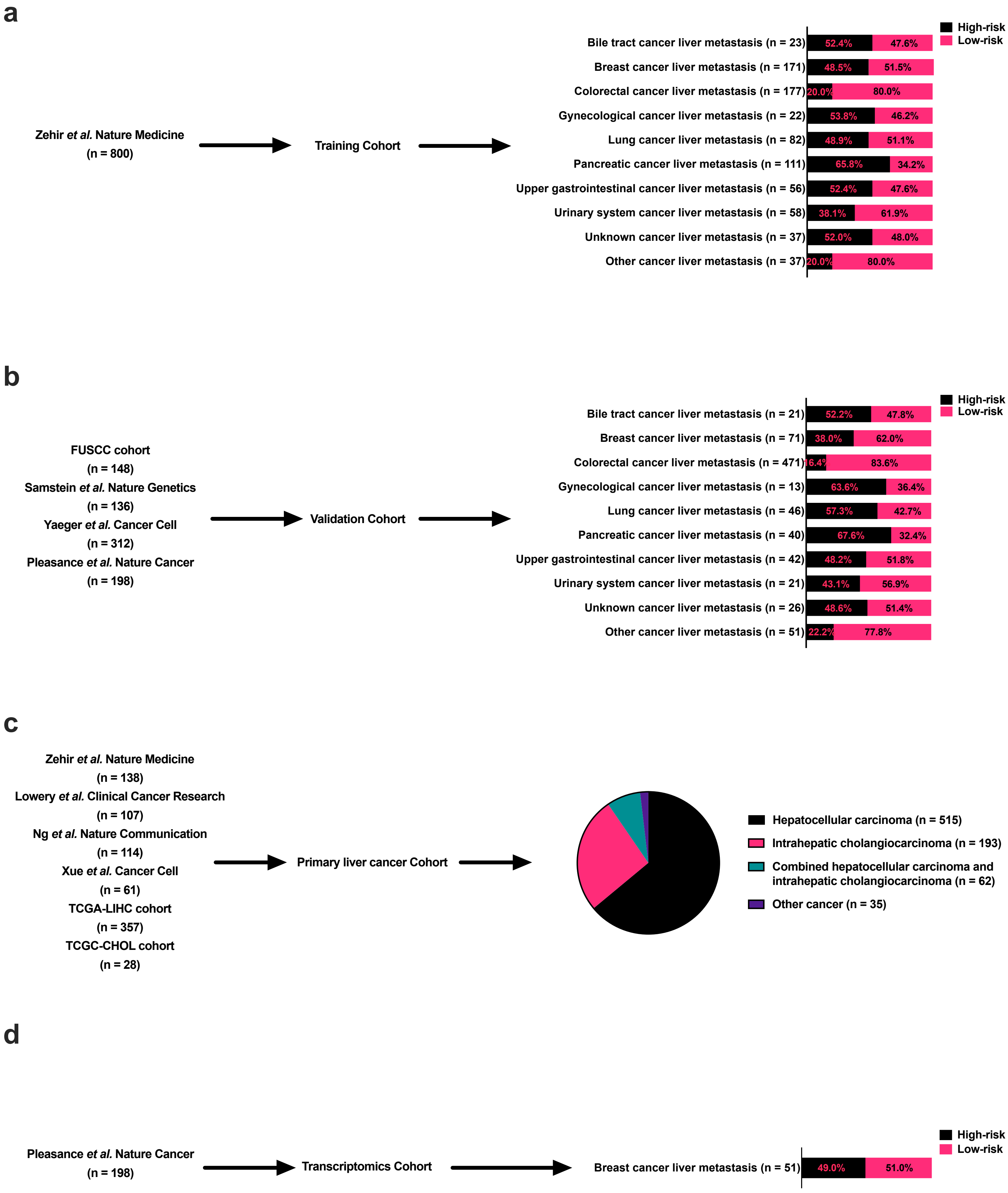

a
Zehir et al. Nature Medicine
(n = 800)
Training Cohort
High-risk
Low-risk
Bile tract cancer liver metastasis (n = 23)
52.4%
47.6%
Breast cancer liver metastasis (n = 171)
48.5%
51.5%
Colorectal cancer liver metastasis (n = 177)
20.0%
80.0%
Gynecological cancer liver metastasis (n = 22)
53.8%
46.2%
Lung cancer liver metastasis (n = 82)
48.9%
51.1%
Pancreatic cancer liver metastasis (n = 111)
65.8%
34.2%
Upper gastrointestinal cancer liver metastasis (n = 56)
52.4%
47.6%
Urinary system cancer liver metastasis (n = 58)
38.1%
61.9%
Unknown cancer liver metastasis (n = 37)
52.0%
48.0%
Other cancer liver metastasis (n = 37)
20.0%
80.0%
b
FUSCC cohort
(n = 148)
Samstein et al. Nature Genetics
(n = 136)
Yaeger et al. Cancer Cell
(n = 312)
Pleasance et al. Nature Cancer
(n = 198)
Validation Cohort
High-risk
Low-risk
Bile tract cancer liver metastasis (n = 21)
52.2%
47.8%
Breast cancer liver metastasis (n = 71)
38.0%
62.0%
Colorectal cancer liver metastasis (n = 471)
16.4%
83.6%
Gynecological cancer liver metastasis (n = 13)
63.6%
36.4%
Lung cancer liver metastasis (n = 46)
57.3%
42.7%
Pancreatic cancer liver metastasis (n = 40)
67.6%
32.4%
Upper gastrointestinal cancer liver metastasis (n = 42)
48.2%
51.8%
Urinary system cancer liver metastasis (n = 21)
43.1%
56.9%
Unknown cancer liver metastasis (n = 26)
48.6%
51.4%
Other cancer liver metastasis (n = 51)
22.2%
77.8%
c
Zehir et al. Nature Medicine
(n = 138)
Lowery et al. Clinical Cancer Research
(n = 107)
Ng et al. Nature Communication
(n = 114)
Xue et al. Cancer Cell
(n = 61)
TCGA-LIHC cohort
(n = 357)
TCGC-CHOL cohort
(n = 28)
Primary liver cancer Cohort
Hepatocellular carcinoma (n = 515)
Intrahepatic cholangiocarcinoma (n = 193)
Combined hepatocellular carcinoma and intrahepatic cholangiocarcinoma (n = 62)
Other cancer (n = 35)
d
Pleasance et al. Nature Cancer
(n = 198)
Transcriptomics Cohort
High-risk
Low-risk
Breast cancer liver metastasis (n = 51)
49.0%
51.0%

File name: Additional file 1 Fig. S1.pdf. **Additional file 1: Fig. S1** The summary of patients included in the training (**a**), validation (**b**) and primary liver cancer (**c**) and transcriptomic (**d**) cohorts.
