# Supplementary material for: A gene mutation-based risk model for prognostic prediction in liver metastases
Source: BMC Genomics. 2023 Aug 26;24:489. doi: 10.1186/s12864-023-09595-9 (PMC10463705; doi:10.1186/s12864-023-09595-9)
Supplement: Supplementary file 1 — Additional file 1: Fig. S1. The summary of patients included in the training (a), validation (b) and primary liver cancer (c) and transcriptomic (d) cohorts. Fig. S2. Assessment of the predictive performance of the gene mutation-based risk model. a. Receiver operating characteristic (ROC) curve of the training cohort. b. ROC curve of the validation cohort. c. Area under the ROC curve (AUC) for different cancer types in the training cohort. d. AUC for different cohorts in the validation cohort. Fig. S3. Assessment of the predictive accuracy of the gene mutation-based risk model. a. Calibration curve for assessing the predictive accuracy of the gene mutation-based risk model in the training cohort. The gray line represents ideal performance. The red line represents actual performance. b. Calibration curve for assessing the predictive accuracy of the gene mutation-based risk model in the validation cohort. The gray line represents ideal performance. The red line represents actual performance. c. Univariate and multivariate Cox regression analyses of gene mutation-based risk model in the training cohort. d. Univariate and multivariate Cox regression analyses of gene mutation-based risk model in the validation cohort. e. Comparison of C-indexes between the gene mutation-based risk model and 10 gene mutations included in the risk model in the training cohort. f. Comparison of C-indexes between the gene mutation-based risk model and 10 gene mutations included in the risk model in the validation cohort. [file 12864_2023_9595_MOESM1_ESM.zip › Additional file 1 Fig. S2.pdf]

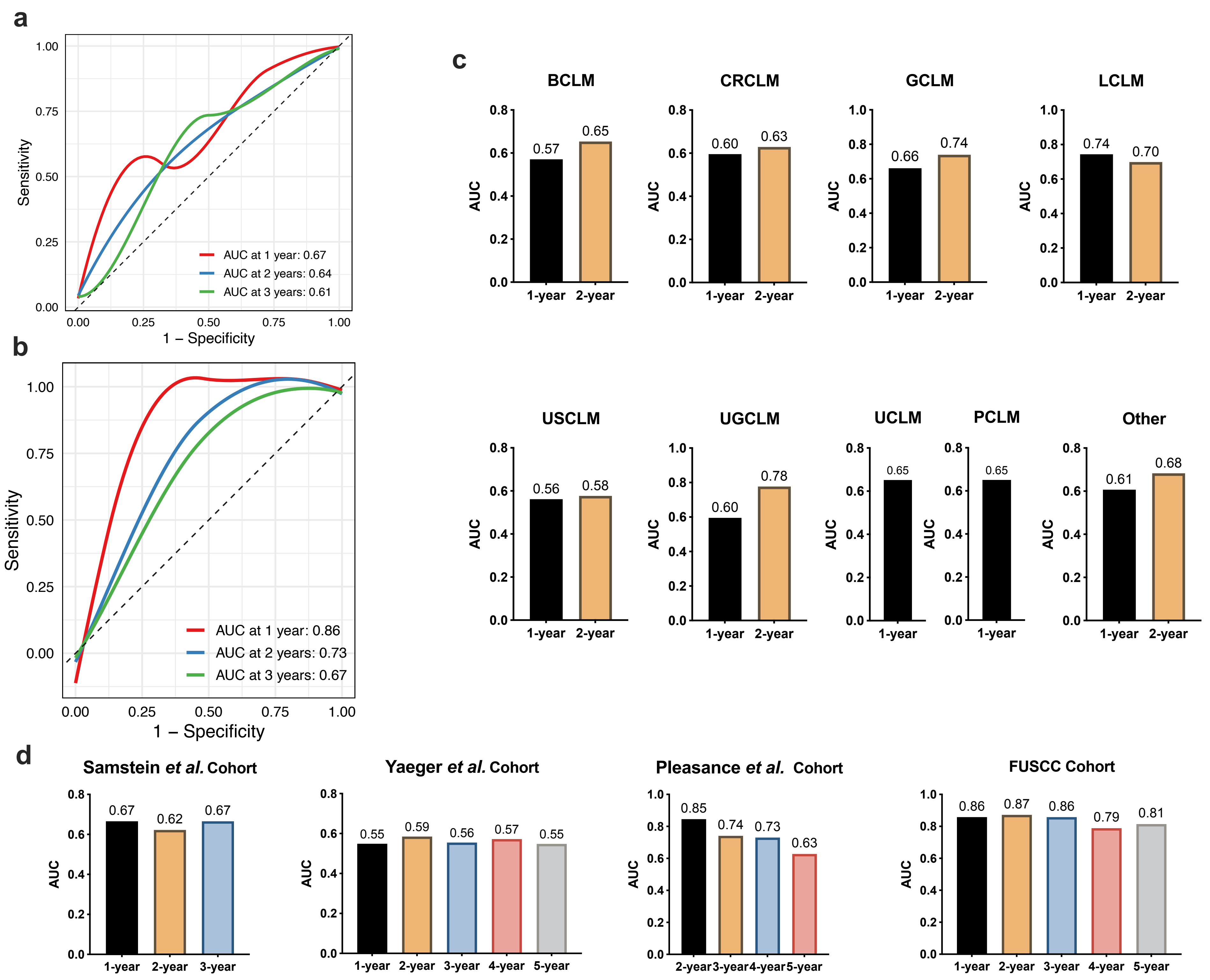
a
Sensitivity
1 – Specificity
AUC at 1 year: 0.67
AUC at 2 years: 0.64
AUC at 3 years: 0.61
b
Sensitivity
1 – Specificity
AUC at 1 year: 0.86
AUC at 2 years: 0.73
AUC at 3 years: 0.67
c
BCLM
0.57
0.65
CRCLM
0.60
0.63
GCLM
0.66
0.74
LCLM
0.74
0.70
USCLM
0.56
0.58
UGCLM
0.60
0.78
UCLM
0.65
PCLM
0.65
Other
0.61
0.68
AUC
1-year
2-year
d
Samstein *et al.* Cohort
0.67
0.62
0.67
1-year
2-year
3-year
Yaeger *et al.* Cohort
0.55
0.59
0.56
0.57
0.55
1-year
2-year
3-year
4-year
5-year
Pleasance *et al.* Cohort
0.85
0.74
0.73
0.63
2-year
3-year
4-year
5-year
FUSCC Cohort
0.86
0.87
0.86
0.79
0.81
1-year
2-year
3-year
4-year
5-year

File name: Additional file 1 Fig. S2.pdf. **Additional file 1: Fig. S2** Assessment of the predictive performance of the gene mutation-based risk model. **a.** Receiver operating characteristic (ROC) curve of the training cohort. **b.** ROC curve of the validation cohort. **c.** Area under the ROC curve (AUC) for different cancer types in the training cohort. **d.** AUC for different cohorts in the validation cohort.
