# Supplementary material for: A gene mutation-based risk model for prognostic prediction in liver metastases
Source: BMC Genomics. 2023 Aug 26;24:489. doi: 10.1186/s12864-023-09595-9 (PMC10463705; doi:10.1186/s12864-023-09595-9)
Supplement: Supplementary file 1 — Additional file 1: Fig. S1. The summary of patients included in the training (a), validation (b) and primary liver cancer (c) and transcriptomic (d) cohorts. Fig. S2. Assessment of the predictive performance of the gene mutation-based risk model. a. Receiver operating characteristic (ROC) curve of the training cohort. b. ROC curve of the validation cohort. c. Area under the ROC curve (AUC) for different cancer types in the training cohort. d. AUC for different cohorts in the validation cohort. Fig. S3. Assessment of the predictive accuracy of the gene mutation-based risk model. a. Calibration curve for assessing the predictive accuracy of the gene mutation-based risk model in the training cohort. The gray line represents ideal performance. The red line represents actual performance. b. Calibration curve for assessing the predictive accuracy of the gene mutation-based risk model in the validation cohort. The gray line represents ideal performance. The red line represents actual performance. c. Univariate and multivariate Cox regression analyses of gene mutation-based risk model in the training cohort. d. Univariate and multivariate Cox regression analyses of gene mutation-based risk model in the validation cohort. e. Comparison of C-indexes between the gene mutation-based risk model and 10 gene mutations included in the risk model in the training cohort. f. Comparison of C-indexes between the gene mutation-based risk model and 10 gene mutations included in the risk model in the validation cohort. [file 12864_2023_9595_MOESM1_ESM.zip › Additional file 1 Fig. S3.pdf]

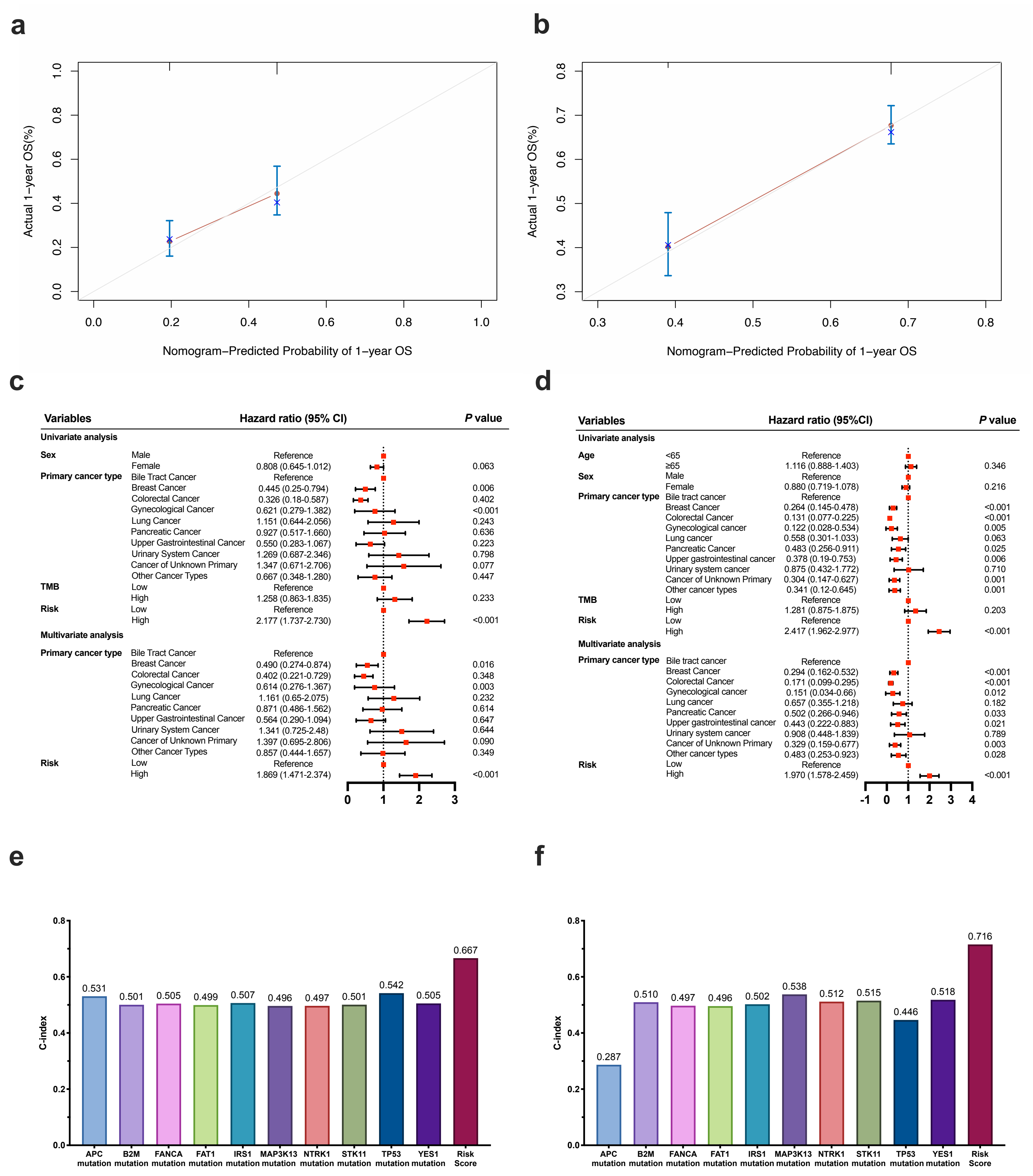
a
Actual 1−year OS(%)
Nomogram−Predicted Probability of 1−year OS
b
Actual 1−year OS(%)
Nomogram−Predicted Probability of 1−year OS
c
Variables
Hazard ratio (95% CI)
P value
Univariate analysis
Sex
Male Reference
Female 0.808 (0.645-1.012) 0.063
Primary cancer type
Bile Tract Cancer Reference
Breast Cancer 0.445 (0.25-0.794) 0.006
Colorectal Cancer 0.326 (0.18-0.587) 0.402
Gynecological Cancer 0.621 (0.279-1.382) <0.001
Lung Cancer 1.151 (0.644-2.056) 0.243
Pancreatic Cancer 0.927 (0.517-1.660) 0.636
Upper Gastrointestinal Cancer 0.550 (0.283-1.067) 0.223
Urinary System Cancer 1.269 (0.687-2.346) 0.798
Cancer of Unknown Primary 1.347 (0.671-2.706) 0.077
Other Cancer Types 0.667 (0.348-1.280) 0.447
TMB
Low Reference
High 1.258 (0.863-1.835) 0.233
Risk
Low Reference
High 2.177 (1.737-2.730) <0.001
Multivariate analysis
Primary cancer type
Bile Tract Cancer Reference
Breast Cancer 0.490 (0.274-0.874) 0.016
Colorectal Cancer 0.402 (0.221-0.729) 0.348
Gynecological Cancer 0.614 (0.276-1.367) 0.003
Lung Cancer 1.161 (0.65-2.075) 0.232
Pancreatic Cancer 0.871 (0.486-1.562) 0.614
Upper Gastrointestinal Cancer 0.564 (0.290-1.094) 0.647
Urinary System Cancer 1.341 (0.725-2.48) 0.644
Cancer of Unknown Primary 1.397 (0.695-2.806) 0.090
Other Cancer Types 0.857 (0.444-1.657) 0.349
Risk
Low Reference
High 1.869 (1.471-2.374) <0.001
d
Variables
Hazard ratio (95%CI)
P value
Univariate analysis
Age
<65 Reference
≥65 1.116 (0.888-1.403) 0.346
Sex
Male Reference
Female 0.880 (0.719-1.078) 0.216
Primary cancer type
Bile tract cancer Reference
Breast Cancer 0.264 (0.145-0.478) <0.001
Colorectal Cancer 0.131 (0.077-0.225) <0.001
Gynecological cancer 0.122 (0.028-0.534) 0.005
Lung cancer 0.558 (0.301-1.033) 0.063
Pancreatic Cancer 0.483 (0.256-0.911) 0.025
Upper gastrointestinal cancer 0.378 (0.19-0.753) 0.006
Urinary system cancer 0.875 (0.432-1.772) 0.710
Cancer of Unknown Primary 0.304 (0.147-0.627) 0.001
Other cancer types 0.341 (0.12-0.645) 0.001
TMB
Low Reference
High 1.281 (0.875-1.875) 0.203
Risk
Low Reference
High 2.417 (1.962-2.977) <0.001
Multivariate analysis
Primary cancer type
Bile tract cancer Reference
Breast Cancer 0.294 (0.162-0.532) <0.001
Colorectal Cancer 0.171 (0.099-0.295) <0.001
Gynecological cancer 0.151 (0.034-0.66) 0.012
Lung cancer 0.657 (0.355-1.218) 0.182
Pancreatic Cancer 0.502 (0.266-0.946) 0.033
Upper gastrointestinal cancer 0.443 (0.222-0.883) 0.021
Urinary system cancer 0.908 (0.448-1.839) 0.789
Cancer of Unknown Primary 0.329 (0.159-0.677) 0.003
Other cancer types 0.483 (0.253-0.923) 0.028
Risk
Low Reference
High 1.970 (1.578-2.459) <0.001
e
C-index
0.531 0.501 0.505 0.499 0.507 0.496 0.497 0.501 0.542 0.505 0.667
APC mutation
B2M mutation
FANCA mutation
FAT1 mutation
IRS1 mutation
MAP3K13 mutation
NTRK1 mutation
STK11 mutation
TP53 mutation
YES1 mutation
Risk Score
f
C-index
0.287 0.510 0.497 0.496 0.502 0.538 0.512 0.515 0.446 0.518 0.716
APC mutation
B2M mutation
FANCA mutation
FAT1 mutation
IRS1 mutation
MAP3K13 mutation
NTRK1 mutation
STK11 mutation
TP53 mutation
YES1 mutation
Risk Score

File name: Additional file 1 Fig. S3.pdf. **Additional file 1: Fig. S3** Assessment of the predictive accuracy of the gene mutation-based risk model. **a.** Calibration curve for assessing the predictive accuracy of the gene mutation-based risk model in the training cohort. The gray line represents ideal performance. The red line represents actual performance. **b.** Calibration curve for assessing the predictive accuracy of the gene mutation-based risk model in the validation cohort. The gray line represents ideal performance. The red line represents actual performance. **c.** Univariate and multivariate Cox regression analyses of gene mutation-based risk model in the training cohort. **d.** Univariate and multivariate Cox regression analyses of gene mutation-based risk model in the validation cohort. **e.** Comparison of C-indexes between the gene mutation-based risk model and 10 gene mutations included in the risk model in the training cohort. **f.** Comparison of C-indexes between the gene mutation-based risk model and 10 gene mutations included in the risk model in the validation cohort.
